# Jaw Dysfunction Is Associated with Neck Disability and Muscle Tenderness in Subjects with and without Chronic Temporomandibular Disorders

**DOI:** 10.1155/2015/512792

**Published:** 2015-03-26

**Authors:** A. Silveira, I. C. Gadotti, S. Armijo-Olivo, D. A. Biasotto-Gonzalez, D. Magee

**Affiliations:** ^1^Alberta Health Services, University of Alberta Hospital, Edmonton, AB, Canada T6G 2B7; ^2^Department of Physical Therapy, Florida International University, Miami, FL 33199, USA; ^3^Faculty of Rehabilitation Medicine, University of Alberta, Edmonton, AB, Canada T6G 2G4; ^4^Department of Physical Therapy and Postgraduate Program in Rehabilitation Sciences, Nove de Julho University, 01504-001 São Paulo, SP, Brazil

## Abstract

*Purpose*. Tender points in the neck are common in patients with temporomandibular disorders (TMD). However, the correlation among neck disability, jaw dysfunction, and muscle tenderness in subjects with TMD still needs further investigation. This study investigated the correlation among neck disability, jaw dysfunction, and muscle tenderness in subjects with and without chronic TMD. *Participants*. Forty females between 19 and 49 years old were included in this study. There were 20 healthy controls and 20 subjects who had chronic TMD and neck disability. *Methods*. Subjects completed the neck disability index and the limitations of daily functions in TMD questionnaires. Tenderness of the masticatory and cervical muscles was measured using an algometer. *Results*. The correlation between jaw disability and neck disability was significantly high (*r* = 0.915, *P* < 0.05). The correlation between level of muscle tenderness in the masticatory and cervical muscles with jaw dysfunction and neck disability showed fair to moderate correlations (*r* = 0.32–0.65). *Conclusion*. High levels of muscle tenderness in upper trapezius and temporalis muscles correlated with high levels of jaw and neck dysfunction. Moreover, high levels of neck disability correlated with high levels of jaw disability. These findings emphasize the importance of considering the neck and its structures when evaluating and treating patients with TMD.

## 1. Introduction

Temporomandibular disorders (TMD) are a musculoskeletal disorder affecting the masticatory muscles, the temporomandibular joint (TMJ), and associated structures. Evidence suggests that TMD are commonly associated with other conditions of the head and neck region, including cervical spine disorders and headache. Presence of neck pain was shown to be associated with TMD 70% of the time [1, 2]. Neuroanatomical and functional connections between masticatory and cervical regions are discussed as explanations for concomitant jaw and neck symptoms [3, 4]. The presence of pain in the masticatory system, especially related to myogenic TMD, could be caused by dysfunctions in the cervical column, or vice versa, showing the intrinsic relationship between the different structures [1, 5].

Although the association of cervical spine disorders and TMD has been studied by different authors, it is far from being exhaustively explained [6, 7]. Most of the studies agree that symptoms from the cervical spine can be referred to the stomatognathic region through the trigeminocervical nucleus. Several studies have examined the presence of signs and symptoms in the cervical region of patients suffering with TMD and that the presence of tender points in the cervical area of these patients is very common [8–13]. de Laat et al. [11] found that, on palpation, 23–67% of the patients with TMD had neck muscle tenderness in the sternocleidomastoid and upper trapezius as well as other cervical and shoulder muscles, which was only rarely present in the control group. Recently, Greenspan et al. [14] measured pressure pain threshold (PPT) in the center of the temporalis, masseter, and trapezius muscles in subjects with and without TMD. They showed that patients with TMD were more sensitive to a wide range of mechanical and thermal pain tests than control subjects, including not only the orofacial area, but also the trapezius muscle.

Muscle tenderness in the cervical spine and jaw was shown to be associated with increased levels of jaw and neck disability. For example, one study by our team revealed a strong relationship between neck disability and jaw disability (*r* = 0.82). A subject with a high level of TMD disability (grade IV) had an increase of about 19 points in the NDI when compared with a person without TMD disability [15]. Disability associated with jaw and neck pain interferes greatly with daily activities and can affect the patient's lifestyle which declines the individual's ability to work and interact in a social environment [6, 8].

Muscle tenderness is the most common sign [8, 16–18] and muscle pain is the most common symptom [19] found in patients with TMD, and their evaluation is still one of the most important methods of establishing a clinical diagnosis of TMD [17, 20], being of particular interest to clinicians treating orofacial pain. Treatment strategies such as exercises, manual therapy, stretching, and education can be targeted to painful and sensitive muscles in order to reduce pain in the orofacial region [8, 20–22].

Although several studies have evaluated neck tenderness in subjects with TMD, none of these studies have evaluated the relationship between the level of tenderness and jaw dysfunction. Moreover, most studies that investigated muscle tenderness in subjects with TMD used palpation techniques, which are difficult to quantify and standardize [10, 11].

There is a great interest on the knowledge for further relationship between stomatognathic system and cervical spine. If further relationship is established, new clinical strategies that target both regions should be considered and, therefore, the need of a multidisciplinary approach should be reinforced in the management of patients with alterations of the stomatognathic system, including TMD patients. In order to further investigate this relationship, the objective of this study was to determine the correlation among neck disability, jaw dysfunction, and muscle tenderness in subjects with chronic TMD. We hypothesized that the higher the level of neck disability, the higher the level of jaw dysfunction and the higher the level of muscle tenderness.

## 2. Methods

### 2.1. Subjects

A convenience sample of 20 female subjects diagnosed with chronic TMD (at least 3-month duration) and 20 healthy female subjects participated in this cross-sectional study. Subjects were recruited from the TMD/Orofacial Pain Clinic at the University of Alberta and by using advertising around the university and on the local television news. Sample size calculation was based on bivariate correlation. Based on a moderated and conservative correlation (*r* = 0.4, effect size) and using *α* = 0.05, *β* = 0.20, and power = 80%, approximately 37 subjects were needed for this study [23].

Subjects with TMD were classified with either myogenous TMD (mainly muscle complaints) or mixed TMD (myogenous and arthrogenous) and presented concurrent neck disability. The subjects were excluded if they presented arthrogenic TMD only, a medical history of neurological, bone, or systemic diseases, cancer, acute pain or dental problems other than TMD, or a history of trauma or surgery to the upper quarter within the last year or if they had taken any pain medication or muscle relaxants less than 4 hours before the diagnostic session.

The healthy group included subjects with no pain or clinical pathology involving the masticatory system or cervical spine for at least one year prior to the start of the study. Exclusion criteria included previous surgery, neurological problems, any acute or chronic musculoskeletal injury, or any systemic diseases that could interfere with the procedure and taking any medication such as pain relieving drugs, muscle relaxants, or anti-inflammatory drugs.

After obtaining consent, all subjects were examined clinically using the research diagnostic criteria for temporomandibular disorders (RDC/TMD) [24] by a physical therapist specialized in TMD. Neck disability was evaluated using the Neck Disability Index (NDI) [25]. The TMD group should score more than 4 points on the NDI in order to be classified as presenting neck disability. To measure their level of jaw disability, all subjects completed the Limitations of Daily Functions in the TMD Questionnaire (LDF-TMDQ) [26]. The healthy group had to score less than 4 points on the Neck Disability Index in order to be considered as having no neck dysfunction.

This study was approved by the Ethics Review Board from the University of Alberta, where the study was conducted.

### 2.2. Questionnaires

The “Limitations of Daily Functions in TMD Questionnaire” (LDF-TMDQ) was used to measure the jaw function of all the subjects in this study. The LDF-TMDQ is multidimensional and includes specific evaluations for TMD patients [26]. The LDF-TMDQ consists of 10 items and 3 factors and these factors are extracted by exploratory factor analysis. The first factor is named “limitation in executing a certain task” and is composed of five items including several problems in daily physical and psychosocial activities; the second factor is called “limitation of mouth opening” which is composed of three items, and the third factor, “limitation of sleeping,” is composed of two items. The internal consistency of the questionnaire was calculated using Cronbach's alpha which was 0.78 for the 10 items, 0.72 for “limitation in executing a certain task,” 0.73 for “limitation of mouth opening,” and 0.77 for “limitation of sleeping,” indicating good consistency. The LDF-TMDQ was tested for concurrent validity with the dental version of the McGill Pain Questionnaire and the authors found correlations ranging between 0.49 and 0.54 [26].

The NDI is a questionnaire designed to give information about how neck pain affects the ability of the subject to manage her everyday life [25, 27–30]. The NDI includes 10 items—7 items are associated with activities of daily living, 2 are linked to pain, and 1 is related to concentration [25, 29]. Each item is scored from 0 (no pain or disability) to 5 (severe pain and disability), and the total score is expressed as a percentage (total possible score = 100%), with higher scores corresponding to greater disability [25, 29]. Depending on the score, the patient was classified as having neck disability or not (0–4 = no disability; 5–14 = mild disability; 15–24 = moderate disability; 25–34 = severe disability; >35 = complete disability) [27]. The NDI has proven to be valid and reliable in measuring neck disability, allowing its use as a guide for clinical-decision making [28–30].

### 2.3. Pressure Pain Threshold (PPT) Measurements

The manual pressure algometer (force dial) was used to measure the muscle tenderness in both groups by one investigator, blinded to the subjects' group allocation. Muscle tenderness was measured bilaterally in the following muscles: masseter (i.e., deep masseter, anterior, and inferior portions of the superficial masseter), temporalis (i.e., anterior temporalis, medial temporalis, and posterior temporalis), sternocleidomastoid, and upper trapezius (i.e., occipital region and half way between C7 and acromion) in a supine position for all muscles but trapezius muscle which was evaluated in seating [17, 31, 32]. These muscles were selected for investigation because previous studies reported that patients with TMD tended to develop tenderness in these muscles [31, 32]. Furthermore, these muscles were easy to evaluate because of their anatomic position, which avoided confusion with other anatomic structures such as joints, ligaments, and other muscles.

**Figure 1:**
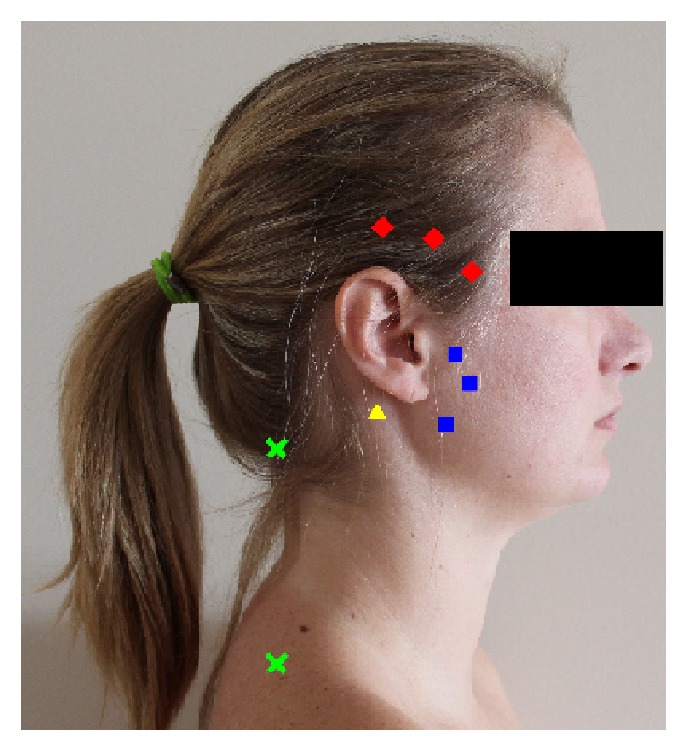
PPT points evaluated (♦ temporalis muscle, ■ masseter muscle, ▲ sternocleidomastoid muscle, and** X** upper trapezius muscle).

The pressure pain threshold (PPT) was defined in this study as the point at which a sensation of pressure changed to pain. At this moment, the subject said “yes,” the algometer was immediately removed, and the PPT was noted [33]. Before the test was performed, the procedure was demonstrated on the investigator's hand and a practice trial was performed on the subject's right hand [33]. During the test, the algometer was held perpendicular to the masticatory (i.e., masseter and temporalis) and neck muscles (i.e., sternocleidomastoid and upper trapezius).  Figure 1 shows the sites in which the muscles were measured. The measurements were repeated 3 times at each site, with 30-second intervals with pressure rate of 1 Kg/sec for the neck muscles and 0.5 Kg/sec for the masticatory muscles [34, 35]. Since the first PPT of a session is usually higher than consecutive measurements, the first PPT measurement was discarded and the mean of the other two PPT measurements was considered to be the final pressure threshold of the sites tested [34].

Pressure rates were decided based on previously studies that showed the most reliable rates to use on cervical and facial muscles [18, 36–38].

### 2.4. Statistical Analysis

Muscle tenderness data for all analyzed muscles, jaw, and neck disability levels were analyzed descriptively. A paired *t*-test was performed to verify whether there were any differences between right and left sides in each pair of muscles. Spearman's rho was used to determine whether there was a correlation among neck disability, jaw dysfunction, and muscle tenderness. The criteria used to interpret the correlation coefficient were as follows: 0.00–0.25: little correlation, 0.26–0.49: low correlation, 0.50–0.69: moderate correlation, 0.70–0.89: high correlation, and 0.90–1.00: very high correlation. The correlation was considered important when the correlation coefficient value was higher than 0.70. The reference values to make this decision were based on values reported by Munro [39].

Level of significance for all statistical analyses was set at *α* = 0.05. The SPSS (SPSS Inc., Chicago), Statistical Program version 18.0 (Statistical Package for the Social Sciences), was used to perform the statistical analysis.

## 3. Results

### 3.1. Subjects Demographics

Mean age for TMD group was 31.05 (SD = 6.9) and for the healthy group was 32.3 (SD = 7.2). Thirteen subjects were classified as having mixed TMD and 7 were classified as having myogenic TMD. The range of neck disability ranged from 0 to 31 (no to severe disability) and the range of jaw dysfunction ranged from 10 to 50 (no to severe disability) among all subjects included in this study.

### 3.2. Correlation between Level of Muscle Tenderness and Jaw Dysfunction and Neck Disability

The correlations (Spearman's rho) between level of muscle tenderness and jaw dysfunction (LDF-TMDQ) as well as between level of muscle tenderness and neck disability (NDI) ranged from low to moderate correlations. Spearman's rho ranged from 0.387 to 0.647 for muscle tenderness and jaw dysfunction and Spearman's rho ranged from 0.319 to 0.554 for muscle tenderness and neck disability (Table 1).

### 3.3. Correlation between Neck Disability and Jaw Dysfunction

It was found that the correlation (Spearman's rho) between jaw disability and neck disability was significantly high (*r* = 0.915, *P* < 0.001). The coefficient of variation was 0.82 indicating that approximately 82% of the variance of jaw disability is explained by the neck disability in this population. Thus, subjects who had no or low levels of jaw disability (evaluated through the JDI) also presented with no or low levels of neck disability (evaluated through the NDI).

## 4. Discussion

This study investigated the correlation among neck disability, jaw dysfunction, and muscle tenderness in subjects with and without chronic TMD.

The main results of this study were that jaw dysfunction and neck disability were strongly correlated, showing that changes in jaw dysfunction might be explained by changes in neck disability and vice versa. Also, the results showed that the higher the level of muscle tenderness in upper trapezius and temporalis muscles is, the higher the level of jaw and neck dysfunction the subject will have. These results add to the body of knowledge in this area providing new information regarding these associations. Furthermore, they corroborated the importance of looking at cervical spine and stomatognathic system as a functional entity when evaluating and treating subjects with TMD, neck pain, and muscle tenderness. Another study that is corroborated to this association was the study by Herpich and colleagues [40], where head and neck posture was found to be different between patients with bruxism and controls. They also found a relationship between posture alterations and the TMD severity.

The discussion will focus on each of the results separately, as well as highlighting the strengths and limitations of this study.

### 4.1. Correlation between Level of Muscle Tenderness of Masticatory and Cervical Muscles and Jaw Dysfunction and Neck Disability

Several studies examined the presence of signs and symptoms in the cervical area of patients suffering with TMD and they have been showing that the presence of tender points in the cervical area of TMD's patients is quite common, which is in line with the findings of this study [8–13]. Both upper trapezius and temporalis muscles had a moderate correlation with jaw dysfunction and neck disability. This finding indicates that increased levels of tenderness in these two muscles were related to higher levels of dysfunction in patients having TMD with concurrent neck disability. Therefore, assessing temporalis and upper trapezius muscles in patients with TMD and concurrent neck disability may allow physical therapists to have a better understanding of the level of dysfunction of these patients and to consider the need of managing these patients as a whole. However, although these results show a trend, moderate correlations just indicate association between levels of dysfunction in patients having TMD and concurrent neck disability with levels of muscle tenderness in both upper trapezius and temporalis muscles [23].

Muscle tenderness is only one factor among multiple factors that could contribute to maintaining or perpetuating a level of dysfunction in people with TMD either in the jaw or in the neck. Usually, jaw dysfunction and neck disability are both related to gender, psychological factors, and social factors. For example, studies have shown that the presence of muscle tenderness is more commonly found in women than in men suffering with signs and symptoms of TMD [8, 41–44]. Females' hormones seem to play a possible etiologic role, since there is a higher prevalence of signs and symptoms of TMD in women than in men as well as a lower prevalence for women in the postmenopausal years [41]. Increased rates of occurrence of TMD have been shown during specific phases of the menstrual cycle and possible adverse effects of oral contraceptives have been cited in the literature [41, 45]. Sherman et al. [45] showed significant differences in terms of pressure pain threshold during different phases of a woman's menstrual cycle. Women who have TMD and have not been using oral contraceptives showed lower pressure pain thresholds during menses and midluteal phases, while women with TMD and using oral contraceptives had stable pressure pain threshold throughout menses, ovulatory, and midluteal phases, with increased intensity at the late luteal phase [45]. Fluctuations in estrogen levels during the menstrual cycle may be related to the level of pressure pain in women [45]. The authors speculated that TMD patients, when exposed to experimental pain stimuli, might benefit from the use of oral contraceptives, since these patients did not experience the same intensity of estrogen depletion levels throughout late luteal and menses phases of the menstrual cycle nor the wide swings in estrogen levels during the ovulation [45].

“Pain is a complex phenomenon influenced by both biologic and psycologic* [sic]* factors” [46] (pp. 236). Younger et al. [47] found several limbic abnormalities in subjects suffering with TMD, showing that these patients had alterations not only in the sensory system, but also within the limbic system. The authors found alterations in the basal ganglia nuclei, which contain neurons responsive to nociceptive input and serve the function of preparing behavioral responses to noxious stimuli. They also found alterations in the anterior insula of patients with TMD. These alterations have been reported to be responsible for the integration of emotional and bodily states [47]. According to the authors, alterations in the anterior insula region appear to be very important in the emotional awareness of internal states and the emotional aspects of the pain experience and anticipation of sensation. It is important to note that pain is also perceived differently by different people, since factors such as fear, anxiety, attention, and expectations of pain can amplify the levels of pain experience [46]. On the other hand, self-confidence, positive emotional state, relaxation, and beliefs that pain is manageable may decrease the sensation of pain [46]. Studies have shown that psychosocial factors are significantly associated with both jaw pain and neck pain [48–50]. Vedolin et al. [50], for example, showed that the PPTs of jaw muscles of patients with TMD were lower throughout a natural stressful event (i.e., academic examination), showing a relationship between stress and anxiety levels with level of muscle tenderness. Another study by Mongini et al. [32] also showed a high relationship between jaw and neck muscle tenderness with the prevalence of anxiety and depression among patients suffering from TMD. Increased levels of stress, anxiety, and depression could enhance sympathetic activity and the release of epinephrine at sympathetic terminals, leading to an increase in acetylcholine activity at the motor endplate. This could start a cascade of events, causing a decreased pressure pain threshold in the muscles [50]. The results of these studies suggest that a more integrated treatment approach including psychosocial assessment is important when treating patients with TMD. Factors that might be related to the development of jaw dysfunction or neck disability were not evaluated in this study, so further conclusions regarding social, emotional, and psychological factors are beyond the scope of this specific study.

### 4.2. Correlation between Neck Disability and Jaw Dysfunction

The correlation (Spearman's rho = 0.915) between jaw disability and neck disability was significantly high in this study. This means that the variance of jaw dysfunction is highly dependent on the neck disability (approximately 82%). Thus, subjects who had high levels of jaw disability (evaluated through the JDI) also presented with high levels of neck disability (evaluated through the NDI) and vice versa. Recently, the study by Armijo-Olivo and colleagues [15] was the first to show the relationship between jaw disability and neck disability. As in the present study, a high correlation between jaw disability and neck disability was found. Until now, the association between neck and jaw was always reported in terms of signs and symptoms, but the authors showed the importance of assessing the impact that the level of disability can have on patients suffering with TMD.

Disability is a complex concept, since it involves more than accounting for the individual signs and symptoms alone. It also includes the perception of the patient about his or her condition as an important factor [15]. The International Classification of Functioning, Disability and Health from the World Health Organization is helping health professionals to understand the importance of viewing chronic pain patients from different perspectives such as body, individual, societal, and environmental [51]. The impact that the disability has on patient's body functions, body structures, activities, and participation shows a more realistic vision of how the disease is impacting an individual's quality of life [15, 51]. TMD patients are a good example of how signs and symptoms can be perceived differently by different individuals. Sometimes severe TMD signs and symptoms may only have a small impact on the quality of life of a patient, while mild signs and symptoms may greatly interfere in other patients' lives. Therefore, assessing the level of disability of patients suffering with TMD is important to have a better view of how this condition is affecting these patients and which treatment approach is best for each situation [15].

The fact that jaw disability and neck disability are strongly related also shows that one has an effect on the other, which provides further information about the importance of assessing and treating both regions when evaluating chronic TMD patients. Assessment of the neck structures such as joints and muscles as well as the disability of patients with TMD could direct clinicians to include the cervical spine in their treatment approach. In addition, if patients with TMD have neck disability in addition to jaw disability, or vice versa, physical therapists and dentists should work together to manage these patients.

As strong correlation between jaw disability and neck disability does not indicate a cause and effect relationship, longitudinal studies where subjects with TMD are followed up to determine the appearance of neck disability are still necessary to determine any cause and effect connection.

### 4.3. Clinical Relevance

This study showed that the higher the level of muscle tenderness, mainly in upper trapezius and temporalis muscles, the higher the level of jaw and neck disability. Therefore, when clinicians assess higher levels of muscle tenderness either in the jaw and/or in the neck regions, they should infer that this could be possibly related to higher levels of jaw and neck disability. This information will guide health professionals to consider new clinical strategies that focus on both masticatory and cervical regions to improve patients' outcomes. Jaw dysfunction and neck disability were strongly correlated, showing that changes in jaw dysfunction might be explained by changes in neck disability and vice versa. This provides further information about the importance of assessing and treating both the jaw and neck regions as a complex system in TMD patients.

### 4.4. Limitations

The convenience sample used increased the potential subject self-selection bias. It was difficult to recognize what characteristics were present in those who offer themselves as subjects, as compared with those who did not, and it was unclear how these attributes might have affected the ability to generalize the outcomes [32]. Although probability samples would have been ideal for this type of study, having accessibility to the general population of TMD patients was limited in this study. Furthermore, even with random selection, not all of the TMD patients who could have been invited to participate in the study would give their consent.

## 5. Conclusions

High levels of muscle tenderness were correlated with high levels of jaw and neck disabilities. Furthermore, jaw dysfunction and neck disability were strongly correlated, showing that changes in jaw dysfunction may be explained by changes in neck disability and vice versa in patients with TMD. This study has highlighted the importance of assessing TMD patients not only at the level of the jaw, but also including the neck region. Muscle tenderness, however, is only one aspect of the TMD. TMD is a complex problem and involves many factors such as gender, levels of anxiety and stress, and the level of socialization of the patient. Future studies investigating the association between neck and jaw should also include factors other than muscle tenderness which are still needed.

## Figures and Tables

**Table 1:** Correlation between muscle tenderness (PPTs) and neck disability and jaw dysfunction.

Spearman's rho			
Side	Muscle	Jaw dysfunction	Neck disability
Right	Temporalis	−0.585	−0.517
	Masseter	−0.512	−0.443
	Sternocleidomastoid	−0.387	−0.319
	Upper trapezius	−0.408	−0.352

Left	Temporalis	−0.646	−0.554
	Masseter	−0.595	−0.48
	Sternocleidomastoid	−0.426	−0.374
	Upper trapezius	−0.647	−0.518
